# Irisin Mitigates Oxidative Stress, Chondrocyte Dysfunction and Osteoarthritis Development through Regulating Mitochondrial Integrity and Autophagy

**DOI:** 10.3390/antiox9090810

**Published:** 2020-09-01

**Authors:** Feng-Sheng Wang, Chung-Wen Kuo, Jih-Yang Ko, Yu-Shan Chen, Shao-Yu Wang, Huei-Jing Ke, Pei-Chen Kuo, Chin-Huei Lee, Jian-Ching Wu, Wen-Bin Lu, Ming-Hong Tai, Holger Jahr, Wei-Shiung Lian

**Affiliations:** 1 Core Laboratory for Phenomics and Diagnostics, Kaohsiung Chang Gung Memorial Hospital, Kaohsiung 83301, Taiwan; wangfs@ms33.hinet.net (F.-S.W.); bulakuo@gmail.com (C.-W.K.); ggyy58720240@gmail.com (Y.-S.C.); vip690221@gmail.com (S.-Y.W.); maggie2258tw@gmail.com (H.-J.K.); bank66882007@gmail.com (P.-C.K.); carol0707@cgmh.org.tw (C.-H.L.); 2 Department of Medical Research, Kaohsiung Chang Gung Memorial Hospital, Kaohsiung 83301, Taiwan; 3 Center for Mitochondrial Research and Medicine, Kaohsiung Chang Gung Memorial Hospital, Kaohsiung 83301, Taiwan; 4 Graduate Institute of Clinical Medical Science, Chang Gung University College of Medicine, Kaohsiung 83301, Taiwan; 5 Department of Orthopedic Surgery, Kaohsiung Chang Gung Memorial Hospital, Kaohsiung 83301, Taiwan; kojy@cgmh.org.tw; 6 Biobank and Tissue Bank, Kaohsiung Chang Gung Memorial Hospital, Kaohsiung 83301, Taiwan; djbluestyle338@hotmail.com; 7 Institute of Biomedical Sciences, National Sun Yat-Sun University, Kaohsiung 804, Taiwan; a0972819661@gmail.com (W.-B.L.); minghongtai@gmail.com (M.-H.T.); 8 Department of Anatomy and Cell Biology, University Hospital RWTH Aachen, 52074 Aachen, Germany; 9 Department of Orthopedic Surgery, Maastricht University Medical Center, 6229 ER Maastricht, The Netherlands

**Keywords:** chondrocyte, irisin, FNDC5, autophagy, mitochondria, osteoarthritis

## Abstract

Compromised autophagy and mitochondrial dysfunction downregulate chondrocytic activity, accelerating the development of osteoarthritis (OA). Irisin, a cleaved form of fibronectin type III domain containing 5 (FNDC5), regulates bone turnover and muscle homeostasis. Little is known about the effect of Irisin on chondrocytes and the development of osteoarthritis. This study revealed that human osteoarthritic articular chondrocytes express decreased level of FNDC5 and autophagosome marker LC3-II but upregulated levels of oxidative DNA damage marker 8-hydroxydeoxyguanosine (8-OHdG) and apoptosis. Intra-articular administration of Irisin further alleviated symptoms of medial meniscus destabilization, like cartilage erosion and synovitis, while improved the gait profiles of the injured legs. Irisin treatment upregulated autophagy, 8-OHdG and apoptosis in chondrocytes of the injured cartilage. In vitro, Irisin improved IL-1β-mediated growth inhibition, loss of specific cartilage markers and glycosaminoglycan production by chondrocytes. Irisin also reversed Sirt3 and UCP-1 pathways, thereby improving mitochondrial membrane potential, ATP production, and catalase to attenuated IL-1β-mediated reactive oxygen radical production, mitochondrial fusion, mitophagy, and autophagosome formation. Taken together, FNDC5 loss in chondrocytes is correlated with human knee OA. Irisin repressed inflammation-mediated oxidative stress and extracellular matrix underproduction through retaining mitochondrial biogenesis, dynamics and autophagic program. Our analyses shed new light on the chondroprotective actions of this myokine, and highlight the remedial effects of Irisin on OA development.

## 1. Introduction

Articular cartilage erosion, together with synovitis and subchondral bone deterioration, are prominent features of knee osteoarthritis (OA) [1]. The chronic degenerative disease accounts for a major etiological cause of joint pain and disability in the elderly [2]. Chondrocyte dysfunction, like survival loss [3], extracellular matrix (ECM) underproduction [4] and proteinase overactivation [5], accelerates the cartilage degradation that deteriorates articular microstructure integrity during OA development. Chronic inflammation, biomechanical disuse and aging increase interleukin (IL)-1, IL-6 and tumor necrosis factor-alpha (TNF-α) production, and escalate oxidative stress, which dysregulates genetic and epigenetic pathways to repress chondrocytic activity and cartilage homeostasis in the OA microenvironment [6].

Expanding evidence reveals that defective organelle machinery impacts chondrocyte fate and metabolism. For example, the loss of mitochondrial biogenesis and dynamics increases chondrocyte apoptosis [7]. Dysfunctional mitochondria result in reactive oxidative radicals overproduction, inducing oxidative damages to protein and DNA stability to hinder ECM anabolism in chondrocytes [8]. Mitochondrial DNA haplogroups are correlated with the risk of knee OA, and also affect oxidative stress and the survival of cybrid cells [9]. The loss of endoplasmic reticulum (ER) chaperones increase ER stress, downregulating cartilage ECM synthesis in osteoarthritic joints [10]. Increased ER stress is present in articular chondrocytes in unilateral anterior cross-bite-mediated OA in mice [11]. The downregulation of ER stress through activating eukaryotic translation initiation factor 2α compromises OA signs, such as cartilage damage, synovium remodeling and osteophyte formation, in anterior cruciate ligament transection-mediated joint injury upon mechanical loading [12]. In addition, autophagy is required to remove unwanted organelles so as to maintain cellular homeostasis through autophagosomal encapsulation and lysosomal degradation of the dysfunctional macromolecules [13]. Decreased autophagy in chondrocytes is correlated with articular cartilage degradation in destabilized medial meniscus (DMM) and age-induced OA knees [14]. Mice lacking autophagy inhibitor mechanistic target of rapamycin kinase (mTOR) in chondrocytes show less articular cartilage damage and chondrocyte apoptosis in DMM-injured knees, as compared to wild-type mice [15]. The molecular mechanism underlying organelle dysfunction in osteoarthritic chondrocyte warrants investigations. 

Fibronectin type III domain containing protein 5 (FNDC5) is a transmembrane protein, composing of an extracellular Irisin domain and a cytosolic C-terminal domain [16]. Peroxisome proliferator-activated receptor gamma coactivator 1α (PGC-1α) signaling transduction activates FNDC5 to increase the secretion of Irisin, which is a soluble peptide with 112 amino acids [16]. Irisin is abundant in skeletal muscle tissue and facilitates the positive impact of moderate exercise on tissue physiology [17,18]. Accumulating studies have revealed the regulatory actions of FNDC5 and Irisin on tissue homeostasis and remodeling. FNDC5 mutant mice show decreased brown fat formation of white adipose tissue upon exercise [19]. Irisin increases the myoblast differentiation of myogenic progenitor cells and promotes the regeneration capacity of notexin-injured skeletal muscle [20]. FNDC5 knockout compromises estrogen deficiency-mediated osteocytic osteolysis and trabecular bone loss [21], as well as downregulating unloading-induced osteocyte apoptosis and osteoporosis development [22]. Irisin recombinant protein increases growth and ECM accumulation in human osteoarthritic chondrocytes [23]. As yet, little is known about FNDC5’s and Irisin’s actions on cartilage integrity in the development of knee OA. 

This study aimed to characterize FNDC5 signaling in human osteoarthritic cartilage and in a mouse model with DMM-mediated OA. We also verified whether Irisin affected mitochondria function or autophagy in chondrocytes and OA development.

## 2. Materials and Methods 

### 2.1. Human Cartilage Specimens

Protocols and experiments for human leftover articular specimens were approved by Institutional Review Board, Chang Gung Memorial Hospital (IRB Affidavit #201901340B0). Upon written informed consent being obtained, 11 patients with end-stage knee OA who required total knee replacement were enrolled. Osteoarthritic tissue and macroscopic healthy specimens lateral to the injured site were harvested during total knee arthroplasty. 

### 2.2. Destabilized Medial Meniscus (DMM)-Induced OA

Animal experiments were approved by Institutional Animal Care and Use Committee, Kaohsiung Chang Gung Memorial (IACUC Affidavit #2019081301). Animals were housed in a specific pathogen-free vivarium with a 12/12 light/dark cycle. Rodent chow and water were provided ad libitum. 12-week-old male C57L/B6 mice were anesthetized by inhaled isoflurane using a Vetflo^®^ anesthetics vaporizer (Kent Scientific Corporation, Torrington, CT, USA). Medial meniscus of the left knee was removed using aseptic surgical procedures. At the end of the experiment, the mice were euthanatized using an overdose of anesthetics. Knee joints were dissected for histological examination. 

### 2.3. Purification of Irisin Recombinant Protein 

DNA coded Irisin (NM027402.4) was cloned and amplified using PCR protocols with primers (forward, 5′-ATTTCATATGAGCCCCTCAGCCCCT-3′; reverse, 5′-CCGCTCGAGCACTCCTTCAT GGTCAC-3′). The clone was ligated in between the NotI and BamHI restriction enzyme sites of pET28a vector (Novagen Inc., Merck KGaA, Darmstadt, Germany). Plasmids were transferred into BL-21SI competent cells. Upon incubating the transformed cells in 3 mM NaCl for 4 h at 30 °C, cells were centrifuged at 6000× *g* and 4 °C for 5 min which was followed by mixing with lysis buffer (pH 8.0) with 20 mM imidazole, 150 mM NaCl, 1 mM EDTA, 1 mM PMSF, 1 μg/mL aprotinin, 1 μg/mL leupeptin and 1 μg/mL pepstatin. The mixtures were homogenized and centrifuged at 9000× *g* and 4 °C for 30 min. Irisin recombinant proteins in the supernatants were mixed with 1 mL Ni-NTA beads for 20 min and eluted through nickel-gel column affinity chromatography (GE Pharmacia, Princeton, NJ, USA) using an elution buffer (pH 7.0) with 20 mM phosphate buffer, 250 mM imidazole and 150 mM NaCl. Irisin recombinant proteins were freeze-dried and stored at −80 °C.

### 2.4. Intra-Articular Injection of Irisin 

20 mg/mL of Irisin recombinant protein were dissolved in normal saline and filtered through 0.22 μm filters. At 1 week postoperatively, a total of 10 μL Irisin mixtures were intra-articularly injected into DMM-injured knees using an insulin needle under the guidance of sonography (10–22 MHz, LOGIO^TM^, GE Healthcare, Princeton, NJ, USA), as previously described [24]. At 8 weeks postoperatively, mice in the sham (*n* = 5), DMM (*n* = 5) and DMM + Irisin (*n* = 5) groups were euthanatized, and knee joints were dissected. 

### 2.5. Gait Profile Analysis

The walking patterns and gait characteristics of injured legs were investigated using the Catwalk analysis system (Noldus Information Technology, Leesburg, VA, USA), as previously described [25]. Footprint histograms were computed using CatWalk software 9.1 (Noldus Information Technology, Leesburg, VA, USA), according to the maker’s instructions. Footprint area (cm^2^), maximum contact area (%), swing speed (cm/s) and duty cycle (%) were calculated using CatWalk XT’s Automatic Footprint Classification software. 

### 2.6. Histomorphometry and Immunohistochemistry

Knee joints between tibiae and femurs were decalcified and embedded in paraffin. Articular cartilage morphology at proximal tibiae was stained using Safranin-O Staining Kits (3-H Biomedical, Uppsala, Sweden), according to the manufacturer’s manuals. The severity of cartilage damage was quantified using the OARSI score. Six sections spanning 200 μm of the specimens were randomly selected for histomorphometry using a microscope with an image analysis unit (Carl Zeiss, Oberkochen, Germany). In a subset of the experiment, apoptotic chondrocytes in sections were stained using TUNEL Assay Kits (ab206386; Abcam, Cambridge, UK). Immunostaining of sections was performed using 8-OHdG (AB5830, Aldrich-Sigma, St. Louis, MO, USA), FNDC5 (ab181884; Abcam, Cambridge, UK), LC3-II (ab48394; Abcam, Cambridge, UK) and UCP-1 (ab10983; Abcam, Cambridge, UK) antibodies. TUNEL-stained and immunostained chondrocytes in each field were counted. Three sections from each mouse and 5 animals were randomly selected for quantification. 

### 2.7. Chondrocyte Cultures 

After euthanasia, articular cartilage from the hips, femurs and tibiae of 7-day-old mice were dissected under a surgical microscope and incubated in 1 unit/mL collagenase (Sigma-Aldrich, St. Louis, MO, USA) to isolate chondrocytes, as previously described [25]. Cells were incubated in DMEM with 10% fetal bovine serum. 5 × 10^5^ cells/well (6-well plates) were incubated in medium with 5 ng/mL IL-1β (R&D Systems, Minneapolis, NE, USA) for 24 h. In total, 5 × 10^5^ chondrocytes were incubated in 1, 5 and 10 ng/mL Irisin recombinant protein for 6 h, followed by incubating in 5 ng/mL IL-1β for another 24 h. In some experiments, the growth of cells (10^4^ cells/well, 96-well plates) was quantified using WST-1 Cell Proliferation Agents, according to the maker’s manual (Sigma-Aldrich, St. Louis, MO, USA).

### 2.8. Assessment of Chondrocytic Activity

For analysis of glycosaminoglycan production, 5 × 10^5^ IL-1β, Irisin, IL-1β + Irisin and vehicle-treated chondrocytes in 20 μL medium were pipetted to form a drop on the culture plates (24-well plates) and incubated in a 5% O_2_, 37 °C humidified chamber for 72 h. Micromass cultures were stained using Alcian Blue Stain Kits (ab150662; Abcam, Cambridge, UK). The stain in the micromass culture was dissolved in 50 μL of 6 M guanidine-HCl, and the absorbance of the mixture was detected using a spectrophotometer at a 620 nm wavelength and normalized with the total proteins of the micromass culture, as previously described [26]. 

### 2.9. Reverse Transcription-Polymerase Chain Reaction (RT-PCR)

The articular cartilage of mouse knee joints and 10^6^ chondrocytes were mixed with TRI Reagent™ Solution (Thermo Fisher Scientific Inc., Waltham, MA, USA) to extract total RNA, according to makers’ manuals. Reverse transcription of 1 μg total RNA was performed using High-Capacity cDNA Reverse Transcription Kits (Thermo Fisher Scientific Inc., Waltham, MA, USA). RT products were mixed with 2× TaqMan^®^ Universal PCR Master Mix and primers for FNDC5 (NC_000070.5; forward, 5′-AATCGTATGGCCTACCTAATG-3′; reverse, 5′-CTGATCTAAGTGCAA TCGA-3′), aggrecan (NC_000073.6; forward, 5′-CGAGTCAACAGCATCTACC-3′; reverse, 5′-GAG TCATTGGAGCGAAGG-3′), collagen II (NC_000081.6; forward, 5′-ACTTTCCTCCGTCTACTG-3′; reverse, 5′-CCTCATCTCTACATCATTGG-5′), SOX9 (NC_000077.6; forward, 5′-GAACGAGAG CGA GAAGAG-3′; reverse, 5′-CTTGAAGATAGCATTAGGAGAG-5′), MMP9 (NC_000068.7; forward, 5′-CAGGCAGGCAGTATCACTCA-3′; reverse, 5′-AGCTCATATGGGTCCGACAG-5′), VEGF (NC_000006.12; 5′-ACACGGTGGTGGAAGAAGAG-3′; reverse, 3′-GGAAGGGAAGATGAG GAAGG-5′), [27,28], Atg4 (NC_000086.7; forward, 5′-CCAGCTTCAGCAAGATCTCC-3′; reverse, 5′-ATACATCCCCAGCCACAGTC-3′), Atg12 (NC_000084.6; forward, 5′-CCAGCCCAA TAGGACTCTTTAAC-3′; reverse, 5′-CACAGCACCGAAATGT CTC-3′), p62 (NC_000077.6; Forward, 5′-GGTGGAGGGTGCTTTGAATA-3′; reverse, 5′-GATGCTGTCCTGGGTTTCT-3′), Mfn1 (NC_000069.6; forward, 5′-ACAGTGGGCTGGAAACTAAATC-‘3; reverse, 5′-GCTGCTAAACGCT CTCTCT-3′), Drp1 (NC_000082.6; forward, 5′-CGGTGGTGCTAGGATTTGTTA-3′; reverse, 5′-ATGGCAGTCAGG ATGTCAATAG-3′), PINK1 (NC_004354.4; forward, 5′-GTGGAATATCTC GGCAGGTT-3′; reverse, 5′-CTCCAT ACTCTCCAGCCAAG-3′), Parkin (NC_000083.6; forward, 5′-TTGCTGGGACGATGTCTTAAT-3′; reverse, 5′-TTGGGTGTGCTCACATTTA-3′) and β-actin (NC_000071.6; forward, 5′-GACGGCCAGGTCATCACTAT-3′; reverse, 3′-CTTCTGCATCCTGTC AGCAA-5′) [29] to quantify threshold values (Ct) for PCR amplification using an ABI 7900 Detection System (Applied Biosystems, Foster City, CA, USA). The relative mRNA expression of interest in specimens was calculated using an equation 2^−ΔΔCt^.

### 2.10. Analysis of Autophagosome and Mitophagosome Formation 

The autophagic vacuoles in cell cultures were probed using Autophagy Detection Kits (Abcam, Cambridge, UK), according to maker’s manuals. In brief, IL-1β, Irisin, IL-1β + Irisin and vehicle-treated chondrocytes (10^3^ cells/slide) were incubated on Falcon™ chambered cell culture slides (Thermo Fisher Scientific Inc., Waltham, MA, USA) for 24 h. Upon removing medium, specimens were rinsed with PBS, fixed with 4% formaldehyde and washed with 1× Assay buffer. The slides were incubated in Green Detection Reagent with fluorescent monodansylcadaverine in the dark for 30 min, and then washed with 1× Assay buffer. The slides were covered with Prolong Gold Antifade Reagent (Cell Signaling Technology, Danvers, MA, USA). In some experiments, mitophagosome was probed using Mitophagy Detection Kits (Dojindo Laboratories, Kumamoto, Japan), according to maker’s instruction. In brief, cells were washed with PBS and incubated in 100 nM Mitophagy Dye Solution for 30 min, followed by incubating cell cultures in medium with IL-1β or Irisin for 24 h. Upon removing the culture medium and washing with PBS, specimens were incubated in 1 mM Lyso Dye Solution for 30 min. Autophagic puncta were evaluated using Olympus Laser Confocal Microscope system. Autophagic vacuoles and mitophagic puncta positive for fluorescence reactions in each cell were counted. In total, 30 cells in each field of each slide were randomly selected.

### 2.11. Mitochondrial Dynamics Assay

Mitochondrial morphology in IL-1β, Irisin, IL-1β + Irisin and vehicle-treated cell cultures (10^3^ cells/slide) was probed using MitoSOX Red (Thermo Fisher Scientific Inc., Waltham, MA, USA) and evaluated using a laser confocal microscope. Mitochondrial morphology was categorized using MicroP software, as previously described [30,31]. Mitochondria with simple, twisted and branched tube-like morphology were counted and categorized via mitochondrial fusion. Globe-like morphologies were counted and considered as mitochondrial fission [30,31]. In total, 20 cells in each field of each slide were randomly selected.

### 2.12. Mitochondrial Membrane Potential, ATP Production and Reactive Oxygen Radical (ROS) Level Assay

IL-1β, Irisin, IL-1β + Irisin, and vehicle-treated chondrocytes (2 × 10^5^ cells) were probed using JC1-1 Mitochondrial Membrane Potential Assay Kits (Abcam, Cambridge, UK). In brief, cells were washed with 1× Dilution Buffer and stained with 20 μM JC-1 at 37 °C in the dark for 15 min. The red (FL2) and green (FL1) fluorescence reactions of each cell were analyzed using BD Accuri C6 flow cytometer (BD Biosciences, San Jose, CA, USA). Mitochondrial membrane potential was calculated from the ratio of FL2/FL1. In some experiments, ATP production of chondrocyte was investigated using ATP Assay Kits (ab83355, Abcam, Cambridge, UK), according to manufacturer’s instructions. In brief, 5 × 10^5^ chondrocytes were homogenized in ATP Assay Buffer. Aliquots of cell lysates were incubated with ATP Reaction Mix for 30 min. Absorbance of the mixtures was quantified using a spectrophotometer at a 570 nm wavelength. ATP levels were calculated from a standard curve plotted by the serial dilution of authentic ATP. The ROS levels in cells were probed using 5-6-chloromethyl-2′,7-dichlorodihydrofluorescein diacetate acetyl ester (CM-H_2_DCFAD; Invitrogen, Thermo Fisher Scientific Inc., Waltham, MA, USA), according to maker’s instructions. In brief, 5 × 10^4^ IL-1β and Irisin-treated cells were incubated in 10 μM CM-H_2_DCFAD at 37 °C for 30 min and subjected to flow cytometry. 

### 2.13. Immunoblotting

Proteins in cell lysates were separated using sodium dodecyl sulfate–polyacrylamide gel electrophoresis. Designated protein bands in the gel were immunoblotted using primary LC3, Atg4, Atg12, UCP-1, Sirt3, SOD2, catalase and actin antibodies together with Thermo Scientific™ SuperSignal™ Western Blotting Kits (Thermo Fisher Scientific Inc., Waltham, MA, USA). Immunoblotted bands were visualized using horseradish peroxidase catalysis of chemiluminescence. Band intensities of LC3-II, Atg4 and Atg12 normalized with band intensity of actin were quantified using a GE ImageQuant LAS4000 system (GE Healthcare-BioSciences AB, Upsala, Sweden).

### 2.14. Statistical Analysis

Differences between the investigations of OA and non-OA in human specimens were analyzed using Wilcoxon test. Differences of analyses among 3 groups in the experimental OA models and cell culture models were analyzed using ANOVA tests and Bonferroni post hoc tests. *p* < 0.05 stood for statistical significance. 

## 3. Results

### 3.1. FNDC5 Loss and Oxidative DNA Damage in Chondrocytes in Human Knee OA

**Figure 1:**
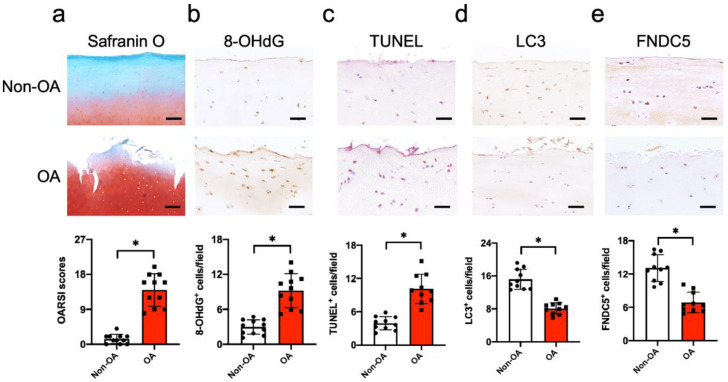
Histological analyses of articular cartilage in human end-stage knee OA. Erosion and fragmentation were present in osteoarthritic cartilage together with significant increases in OARSI scores (**a**); scale bar, 40 μm. Increased chondrocytes showed 8-OHdG immunostaining (**b**) and TUNEL staining (**c**) (scale bar, 20 μm), whereas few chondrocytes displayed LC3 (**d**) and FNDC5 immunostaining (**e**); scale bar, 20 μm. Articular cartilage damage was quantified using OARSI scales. TUNEL, LC3-II and FNDC5 were probed using immunohistochemistry. Data are mean ± standard errors calculated from 11 patients. Asterisks * indicate significant differences from the non-OA group. OA, osteoarthritic cartilage; non-OA, healthy cartilage.

We examined whether FNDC5 signaling was changed in articular chondrocytes in human knee OA. Articular biopsies were harvested from end-stage knee OA during total knee arthroplasty. The OA site showed cartilage erosion and fragmentation histopathology, as compared to the healthy site. The severity of cartilage damage, as evident from OARSI scores, was significantly increased in the OA group (Figure 1a). A great number of chondrocytes in the osteoarthritic cartilage showed strong 8-hydroxydeoxyguanosine (8-OHdG) immunostaining (Figure 1b) and TUNEL staining (Figure 1c), together with weak autophagy marker LC3-II immunostaining (Figure 1d), which suggested that oxidative damage, apoptosis and defective autophagy were present in the OA group. Few chondrocytes in the osteoarthritic cartilage showed FNDC5 immunoreaction, as compared to the healthy cartilage (Figure 1e).

### 3.2. Irisin Attenuated Articular Cartilage Loss and Irregular Gait of DMM-Injured Knees

**Figure 2:**
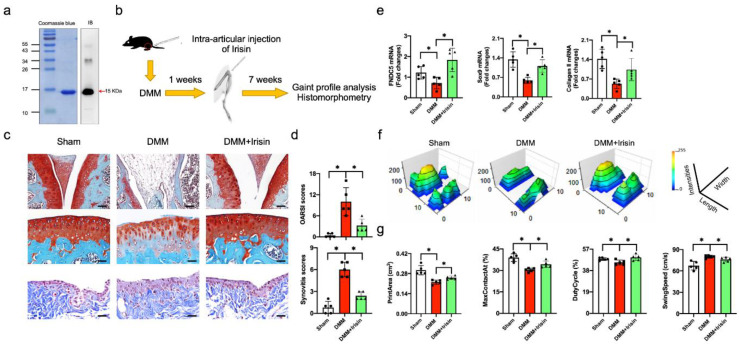
Analyses of Irisin actions to OA development and gait profiles of DMM-injured knee joints. Purified recombinant proteins showed Irisin immunoreactivity (**a**). Schematic drawing for intra-articular injection of Irisin recombinant protein in DMM-injured knee joints (**b**). Irisin treatment compromised cartilage destruction and synovitis in DMM-injured knees (**c**) (upper panel scale bar, 160 μm; middle and lower panels scale bar, 40 μm), as well as reduced OARSI and synovitis scores (**d**). Irisin improved the DMM-induced loss of FNDC5, Sox9 and collagen II in injured knee joints (**e**). DMM-mediated irregular footprint histograms were reversed upon Irisin treatment (**f**). Irisin attenuated DMM-induced dysregulated footprint area, maximum foot contact, duty cycle and swing speed (**g**). Joint damage, chondrocyte markers and gait profiles were probed using histomorphometry, RT-qPCR and the Catwalk system. Data are mean ± standard errors calculated from 5 mice. Asterisks * indicate significant differences between groups.

Irisin is a soluble peptide cleaved from the extracellular domain of FNDC5, regulating the apoptotic program or autophagic reaction in various cell types [23,32]. The analyses of decreased FNDC5 signaling in osteoarthritic chondrocytes in human OA prompted us to isolate Irisin recombinant proteins and verify whether the gained Irisin function changed knee OA development. We constructed expression vector coding for Irisin and isolated the recombinant protein corresponding to 15 kDa. Immunoblotting confirmed the Irisin immunoactivity of the recombinant protein (Figure 2a). We adopted the destabilization of medial meniscus (DMM) in the knee joints of mice as a well-established in vivo model of OA development [33]. The recombinant protein was intra-articularly injected into DMM-injured knee joints 1 week postoperatively (Figure 2b). DMM-injured joints showed articular cartilage degradation, synovium hypertrophy and subchondral bone exposure histopathology, as compared to the sham group 8 weeks postoperatively. The Irisin-treated joints displayed less cartilage injury and synovitis than the DMM group (Figure 2c). Likewise, the severity of articular cartilage damage and synovitis was significantly increased in the DMM group, as evidenced in OARSI and synovitis scores, which were improved in the Irisin-treated group (Figure 2d). Consistent with the histopathological analyses, DMM resulted in significant losses of FNDC5, extracellular matrix collagen II and chondrocytic transcription factor Sox9, as evidenced in RT-PCR analyses. Irisin treatment reversed these molecules in the DMM-injured joints (Figure 2e).

The dysregulated joint kinematic characteristic is a prominent feature of OA-induced joint dysfunction. We adopted a catwalk analysis approach to verify whether Irisin treatment changed gait profiles. The DMM-injured legs showed irregular footprints, which were improved in the Irisin-treated group (Figure 2f). Significantly decreased footprint area (cm^2^), maximum footprint contact (%) and duty cycle (%) and increased swing speed (cm/s) were present in DMM-injured legs. Irisin treatment significantly attenuated the DMM-mediated dysregulation of footprints and walking patterns (Figure 2g).

### 3.3. Irisin Attenuated DMM-Induced Defective Autophagy and Survival in Chondrocytes

**Figure 3:**
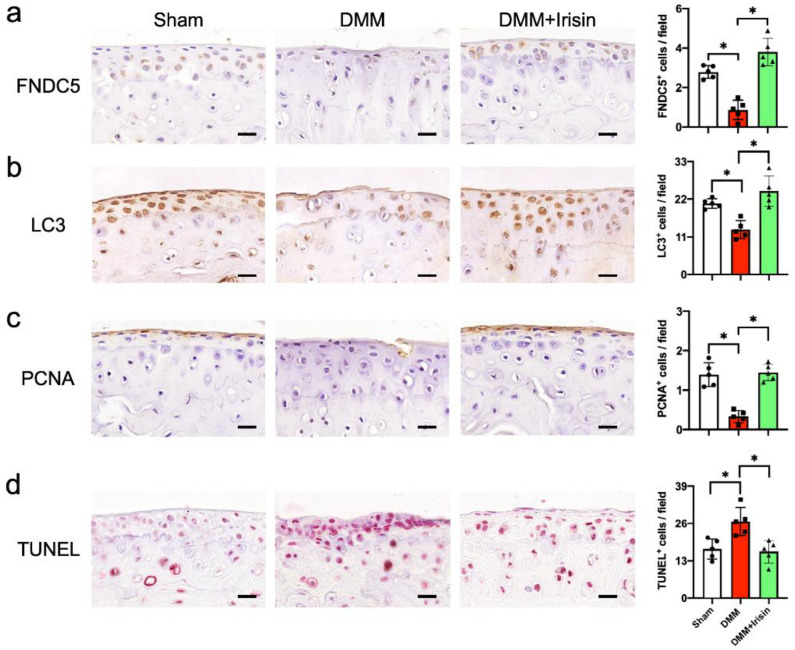
Immunohistochemical analyses of articular cartilage in sham and DMM-injured knees. Few chondrocytes showed FNDC5 (**a**), LC3 (**b**) or PCNA (**c**) immunostaining in DMM-injured cartilage (scale bar, 20 μm), whereas increased chondrocytes displayed TUNEL staining (**d**); scale bar, 20 μm. FNDC5, LC3 and PCNA immunoreactions were reversed and TUNEL staining was downregulated in Irisin-treated knee joints. Data are mean ± standard errors calculated from 5 mice. Asterisks * indicate significant differences between groups.

We conducted immunohistochemical analysis to examine if Irisin signaling affected survival or autophagy in chondrocytes in an OA microenvironment. Few chondrocytes in the DMM-injured articular cartilage showed FNDC5 (Figure 3a) or LC3 (Figure 3b) immunostaining together with the weak cell growth marker proliferating cell nuclear antigen (PCNA) immunoreaction (Figure 3c), whereas plenty of chondrocytes displayed TUNEL staining (Figure 3d). The FNDC5, LC3 and PCNA immunoreactions in chondrocytes were reversed in the Irisin-treated joints. The DMM-mediated upregulation of TUNEL staining was also repressed upon Irisin treatment.

### 3.4. Irisin Alleviated ECM Production of Inflamed Chondrocytes

**Figure 4:**
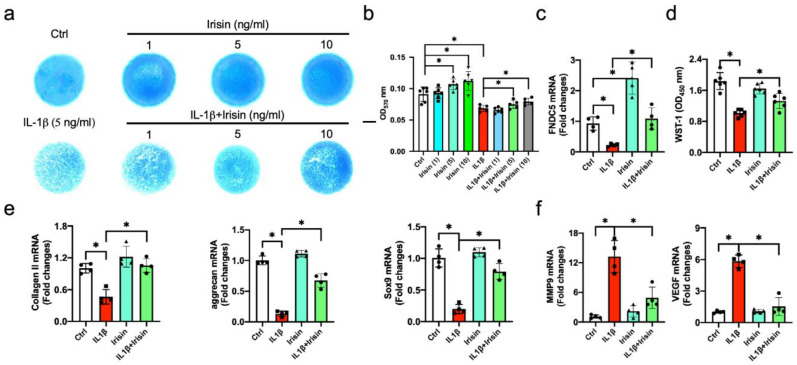
Analyses of growth and ECM production of IL-1β and Irisin-treated chondrocytes. Alcian blue-stained glycosaminoglycan production in IL-1β and Irisin-treated chondrocyte micromass cultures (**a**); scale bars, 5 mm. Irisin dose-dependently improved ECM production of inflamed chondrocyte micromass (**b**). Irisin reversed IL-1β-mediated loss of FNDC5 (**c**), cell growth (**d**), collagen II, aggrecan and Sox9 expression (**e**), as well as repressing MMP9 and VEGF (**f**) expression. Glycosaminoglycan production, cell growth and chondrocyte markers were probed using Alcian blue staining, WST-1 uptake, and RT-qPCR. Data are mean ± standard errors calculated from 4–6 experiments. Asterisks * indicate significant differences between groups.

The investigations of Irisin-mediated repression of articular cartilage destruction in the development of OA prompted us to characterize organelle machinery by which Irisin promoted chondrocyte survival of the injured cartilage. Chondrocytes were incubated in 5 ng/mL IL-1β as an in vitro model simulating inflamed chondrocytes in the OA microenvironment [34]. IL-1β significantly decreased Alcian blue-stained glycosaminoglycan production of chondrocyte micromass cultures 72 h after incubation (Figure 4a). Irisin dose-dependently promoted ECM accumulation above the baseline, as well as attenuating ECM underproduction in the inflamed chondrocytes (Figure 4b). The treatment with 10 ng/mL Irisin had the greatest promotion and was chosen for succeeding experiments. IL-1β significantly decreased FNDC5 expression (Figure 4c) and cell growth after 24 h of incubation (Figure 4d). Chondrocytic markers collagen II, aggrecan and Sox9 were significantly downregulated in the inflamed chondrocytes (Figure 4e), whereas synovitis-promoting factors matrix metalloproteinase 9 (MMP9) and vascular endothelium growth factor (VEGF) (Figure 4f) were increased. Irisin significantly reversed the IL-1β-mediated loss of FNDC5 signaling, proliferation capacity and chondrocytic markers, as well as repressing MMP9 and VEGF expression.

### 3.5. Irisin Improved Autophagy and Apoptosis in Inflamed Chondrocytes

**Figure 5:**
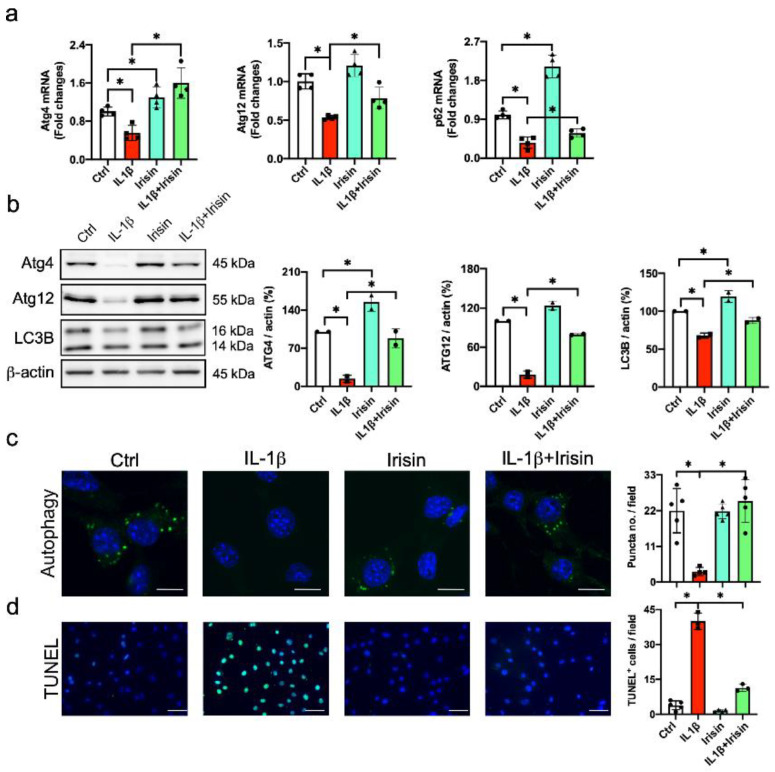
Analyses of autophagy and apoptosis in IL-1β and Irisin-treated chondrocytes. Irisin repressed IL-1β-mediated loss of Atg4, Atg12 and p62 (**a**), as well as improving LC3-II conversion (**b**). Irisin improved the IL-1β-induced loss of monodansylcadaverin-stained autophagic puncta formation (**c**) (scale bar, 10 μm) and compromised apoptosis (**d**) (scale bar, 30 μm) in chondrocytes. The autophagic markers, autophagic puncta and apoptosis in chondrocytes were probed using RT-qPCR, laser confocal microscopy and TUNEL staining. Data are mean ± standard errors calculated from 4–5 experiments. Asterisks * indicate significant differences between groups.

Chronic inflammation dysregulates autophagy, impairing survival and ECM anabolism to accelerate OA development [7,8]. We investigated whether Irisin altered the autophagic or mitochondrial machinery in inflamed chondrocytes. IL-1β significantly decreased autophagic markers Atg4, Atg12 and p62 expression (Figure 5a), as well as inhibiting LC3-II levels. Irisin significantly attenuated autophagic markers and LC3-II conversion in the inflamed chondrocytes (Figure 5b). In addition, plenty of autophagic vacuoles (evident from the fluorescent monodansylcadaverin staining) were present in the cytoplasmic compartment in the vehicle-treated chondrocytes, whereas IL-1β-treated cells showed poor autophagic puncta formation. The IL-1β-induced autophagic vesicle loss was significantly downregulated in the Irisin-treated chondrocytes (Figure 5c). In addition, IL-1β-treated chondrocytes showed increased apoptosis, as evident from the fluorescent TUNEL staining. This effect was significantly downregulated in the Irisin-treated group (Figure 5d).

### 3.6. Irisin Preserved Mitochondrial Fusion and Mitophagy in Inflamed Chondrocytes

**Figure 6:**
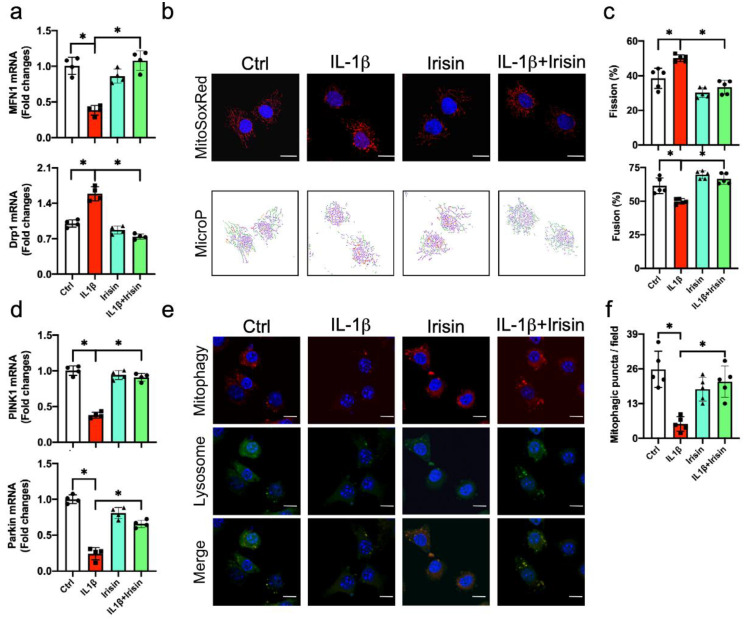
Analyses of mitochondrial dynamics and mitophagy in chondrocytes. Irisin reversed the IL-1β-mediated suppression of Mfn1, Drp1 (**a**), MitoSoxRed-stained morphology (**b**) (scale bar, 10 μm) and mitochondrial fusion (**c**), as well as improving PINK1, Parkin (**d**), Mitophagy dye-stained mitophagic puncta (**e**) and mitophagosome formation (**f**); scale bar, 10 μm. Mitochondrial dynamics markers, mitochondrial morphology, mitophagy markers and mitophagosome were probed using RT-qPCR, fluorescence MitoSoxRed and fluorescence Mitophagy dye together with Lyso dye. Data are mean ± standard errors calculated from 4–5 experiments. Asterisks * indicate significant differences between groups.

The deregulated autophagy of mitochondria (mitophagy) in chondrocytes accelerates OA development [35]. Given that defective autophagy was present in inflamed chondrocytes, we verified whether Irisin signaling affected mitochondrial machinery in inflamed chondrocytes. IL-1β significantly decreased the mitochondrial fusion marker mitofusin 1 (Mfn1), whereas the mitochondrial fission marker dynamin related protein 1 (Drp1) was increased (Figure 6a). We adopted the MitoSox Red probe together with the MicroP image analysis approach to characterize whether IL-1β or Irisin changed mitochondrial dynamics. Mitochondria with simple, twisted and branch tube-like shapes were present in a fusion state. Globe-shape mitochondria were a feature of the fission reaction (Figure 6b). IL-1β significantly reduced the mitochondrial fusion, whereas the mitochondrial fission reaction was upregulated. These effects were reversed in the Irisin group (Figure 6c). In addition, IL-1β significantly decreased mitophagy markers phosphatase and tensin homologue-induced putative kinase 1 (PINK1) and Parkin expression (Figure 6d). Mitophagic puncta showing fluorescent reactions with green Mitophagy dye and red Lyso dye were present in the cytoplasmic compartments in chondrocytes (Figure 6e). IL-1β-treated cells displayed poor mitophagosome formation (Figure 6f). Irisin treatment attenuated the IL-1β-induced loss of PINK1 and Parkin expression, as well as improving mitophagic vacuole development and mitophagosome formation.

### 3.7. Irisin Protected from UCP-1, Sirt3, Catalase Loss and Mitochondrial Dysfunction

**Figure 7:**
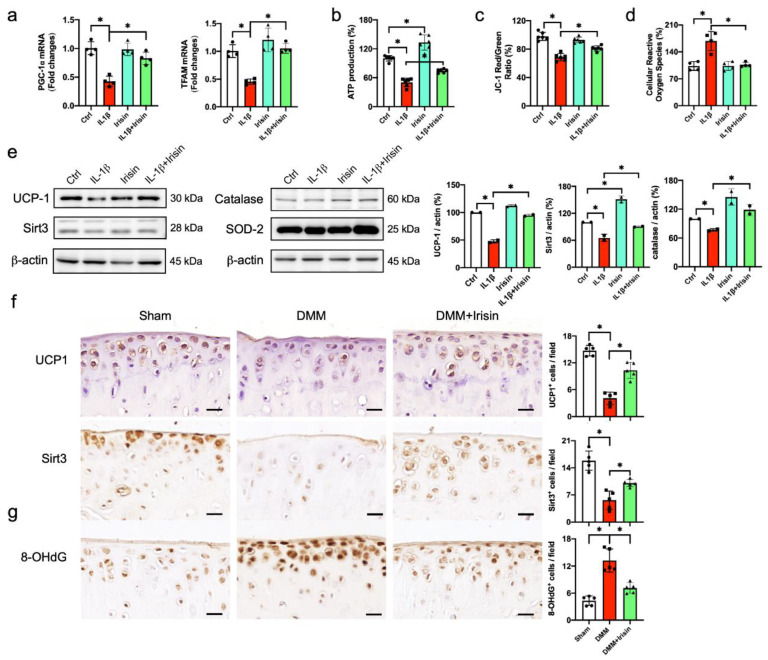
Analyses of mitochondrial regulators and antioxidants in chondrocytes. Irisin attenuated IL-1β-induced loss of PGC-1α and Tfam expression (**a**), ATP production (**b**), membrane potential depolarization (**c**) and reactive oxygen species production (**d**), as well as reversing UCP-1, Sirt3 and catalase protein expression (**e**). Irisin improved Sirt3 and UCP-1 (**f**), whereas 8-OHdG (**g**) immunostaining was increased in articular chondrocytes in DMM-injured knees (8 scale bar, 20 μm). Mitochondrial markers, ATP production, membrane potential and reactive oxygen species were probed using RT-qPCR, Mitochondrial ATP kits, JC-1 probe. Data are mean ± standard errors calculated from 4–5 experiments. Asterisks * indicate significant differences between groups.

IL-1β significantly inhibited mitochondrial biogenesis, like peroxisome-proliferator-activated receptor-γ coactivator-1α (PGC-1α), mitochondrial transcription factor (Tfam) (Figure 7a) and ATP production (Figure 7b), as well as disrupting mitochondrial membrane potential (Figure 7c) and increasing reactive oxygen production (Figure 7d). Irisin treatment significantly improved PGC-1α, Tfam loss and ATP underproduction, preserving membrane potential in inflamed chondrocytes. Sirtuin 3 (Sirt3) and uncoupling protein-1 (UCP-1) signaling are indispensable in mitochondrial integrity and biogenesis [36]. IL-1β inhibited Sirt3 and UCP-1 signaling together with catalase underproduction. These mitochondrial regulators and antioxidants were improved in the Irisin-treated cells. IL-1β or Irisin did not significantly affect SOD2 levels in chondrocytes (Figure 7e). Likewise, few chondrocytes in the DMM-injured cartilage showed Sirt3 and UCP-1 immunostaining, as compared to the sham group. The DMM-mediated loss of the Sirt3 and UCP-1 immunoreactions was reversed upon Irisin treatment (Figure 7f). Intra-articular administration with the Irisin recombinant protein also attenuated oxidative damage marker 8-OHdG immunostaining in chondrocytes in DMM-injured cartilage (Figure 7g).

## 4. Discussion

FNDC5 signaling components play an important role in mediating the beneficial effects that physical activity and moderate exercise evoke, maintaining intracellular homeostasis and metabolism so as to stifle senescence programs, degeneration processes and inflammatory reactions in various tissues in aging, overweight and diabetic conditions [37]. The myokine also communicates via the muscle-bone axis pathway to regulate bone mineral acquisition and osteoporosis development [38], as well as orchestrating muscle-kidney interplay to control renal anabolism and nephropathy [39]. Of interest, regular moderate exercise is found to alleviate joint pain and severity of OA, improving the kinematic activity of injured knees and hips [1]. Given that physical exercise facilitates cartilage homeostasis, the analyses of FNDC5 loss together with the chondrocyte apoptosis revealed in human end-stage knee osteoarthritic cartilage prompted us to understand what role the myokine may play in chondrocyte survival and cartilage integrity in OA. This study revealed that Irisin signaling was required to protect against oxidative damage, apoptosis and ECM underproduction in inflamed chondrocytes, delaying OA development in DMM-injured knees. The investigations into the Irisin upregulation of chondrocytic activity were in agreement with a study showing that Irisin promotes the ECM production of chondrocytes harvested from human osteoarthritic cartilage [23]. Collective analyses also offered productive insight into the Irisin stabilization of mitochondrial dynamics, biogenesis and autophagy in inflamed chondrocytes. This study sheds new light on the subcellular organelle and antioxidative mechanism by which FNDC5 signaling represses chondrocyte dysfunction in the development of OA, as well as highlighting the remedial effects of Irisin recombinant protein on cartilage injury and osteoarthritic disorders.

The histopathological features were that defective autophagy and oxidative damage in chondrocytes were present in patients with end-stage knee OA and in mice with DMM-mediated knee joint injury. The analyses were in agreement with other groups’ studies showing that oxidative stress [40] and autophagy loss [8,41] are correlated with the severity and development of human knee OA. Autophagy failure escalates apoptotic programs in osteoarthritic chondrocytes; however, very little is known about the regulatory mechanism underlying these deleterious reactions. Increasing studies have shown that FNDC5 signaling components are important for stabilizing autophagic reactions that shield from tissue deterioration. For example, hepatocyte-specific FNDC5 knockout mice show defective autophagy, hyperlipidemia and fatty livers [42]. Irisin attenuates pressure overload-inhibited autophagy in cardiomyocytes and hypertrophy in transverse aortic constriction-injured hearts [43]. This study discovered that gains in Irisin signaling reversed autophagic influx together with autophagosome formation, compromising apoptosis to maintain survival and ECM production in the inflamed chondrocytes. Profound findings of improved autophagy, survival and ECM anabolism in Irisin-treated chondrocytes hinted that the myokine may impact mitochondrial function to facilitate chondrocyte metabolism.

FNDC5 and Irisin signaling induce repressing effects on mitochondrial dysfunction-mediated oxidative stress in ischemia/reperfusion-injured hepatic tissue [44], as well as protecting pulmonary tissue from the ischemia/reperfusion-induced loss of mitochondrial machinery and biogenesis [45]. The loss of FNDC5 function disrupts mitochondrial ATP production and membrane integrity, hindering the differentiation of embryonic stem cells into cardiac lineages [46]. This study revealed that Irisin sustained a plethora of mitochondrial activities, like Tfam transcription, ATP synthesis, membrane potential and reactive oxygen species production, etc., compromising apoptosis and ECM underproduction in the inflamed chondrocytes. Moreover, dysregulated mitochondrial dynamics with fusion and fission reactions mediate tissue degeneration. Increased mitochondrial fission accelerates apoptotic programs [47]. MFN2 signaling is increased in senescent chondrocytes and osteoarthritic cartilage. Forced MFN2 expression worsens DMM-mediated articular cartilage injury, whereas knocking down the molecule delays OA conditions [48]. The investigations into Irisin downregulation of the mitochondrial fission reaction in inflamed chondrocytes also explained the protective actions of the myokine against mitochondrial dysfunction. 

Irisin signaling reversed the crosstalk of autophagy and the mitochondrial machinery, as the mitophagic reaction, like the autophagic markers PINK1 and Parkin together with mitophagosome formation, was improved in chondrocytes under inflammatory stress. The role of mitophagy in chondrocytes during osteoarthritis remains uncertain. Increased PINK and autophagic markers are present in human osteoarthritic chondrocytes. The knockdown of PINK signaling attenuates monosodium iodoacetate-induced articular damage and OA development [49]. Ansari et al. reveal that Parkin signaling promotes the clearance of dysfunctional mitochondrial and wards off apoptosis of osteoarthritic chondrocytes [50]. Increased mitophagy together with stable mitochondrial biogenesis attenuate obesity-induced OA upon adenosine A2A receptor treatment [51]. This study showed that improved mitophagy was correlated with increased survival and ECM production in inflamed chondrocytes upon Irisin treatment. We speculated that mitophagy contributes to chondrocytic activity, and cartilage integrity may depend on extracellular stimulation or OA models. 

The protective effects on the mitochondria in chondrocytes that Irisin signaling facilitated did prompt us to verify the molecular mechanism underlying these events. Accumulating studies show that PCG-1α, UCP-1 and Sirt3 signaling are master regulators, indispensable in mitochondrial function, biogenesis and autophagy [52,53]. These mitochondrial regulators also mediate the Irisin promotion of tissue homeostasis [17,39]. Chronic IL-1β stress interrupts PGC-1α signaling-mediated mitochondrial membrane potential and ATP production in chondrocytes [54], as well as decreasing the UCP-1 level in brown adipocytes via the Sirt pathway [55]. This study uncovered that Irisin reversed PCG-1α, UCP-1 and Sirt3, together with antioxidant catalase, to improve membrane potential and mitochondrial biogenesis in the IL-1β-stressed chondrocytes. Collective evidence sheds new light onto the molecular mechanism underlying the Irisin repression of mitochondrial dysfunction and oxidative stress in chondrocytes during OA development. 

The protective actions of FNDC5 signaling components towards inflamed chondrocyte stood true for slowing OA development, as the intra-articular injection of the Irisin recombinant protein that we purified improved survival and autophagy in articular chondrocytes in DMM-injured knees, as well as compromising a plethora of OA signs, like oxidative stress, articular degradation and synovitis. The analyses of improved walking patterns and gait characteristics of DMM-injured legs underpinned the evidence of Irisin protection against joint microstructure damage during OA development. While FNDC5 loss was present in human osteoarthritic cartilage, the limitation of this study is that the Irisin level in the development of the joint disorder warrants investigations. This study conveys a productive insight into the remedial potential of Irisin recombinant protein for subchondral organelle integrity during knee OA development. 

## 5. Conclusions

Taken together, FNDC5 signaling loss was correlated with chondrocyte apoptosis during OA knee development. Irisin repressed defective autophagy, mitochondria dysfunction and mitophagy failure, improving survival and ECM anabolism in inflamed chondrocytes through promoting UCP-1 and Sirt3 signaling. This study delivers a new insight into the chondroprotective mechanism by which Irisin protects against chondrocyte dysfunction, and highlights the remedial potential of Irisin recombinant protein for OA disease.
